# Effect of Long-Term Zinc Pollution on Soil Microbial Community Resistance to Repeated Contamination

**DOI:** 10.1007/s00128-012-0523-0

**Published:** 2012-01-26

**Authors:** Beata Klimek

**Affiliations:** Institute of Environmental Sciences, Jagiellonian University, Gronostajowa 7, 30-387 Kraków, Poland

**Keywords:** Combined stressors, Trace metals, Soil respiration rate, Soil pollution

## Abstract

The aim of the study was to compare the effects of stress (contamination trials) on the microorganisms in zinc-polluted soil (5,018 mg Zn kg^−1^ soil dry weight) and unpolluted soil (141 mg Zn kg^−1^ soil dw), measured as soil respiration rate. In the laboratory, soils were subjected to copper contamination (0, 500, 1,500 and 4,500 mg kg^−1^ soil dw), and then a bactericide (oxytetracycline) combined with a fungicide (captan) along with glucose (10 mg g^−1^ soil dw each) were added. There was a highly significant effect of soil type, copper treatment and oxytetracycline/captan treatment. The initial respiration rate of chronically zinc-polluted soil was higher than that of unpolluted soil, but in the copper treatment it showed a greater decline. Microorganisms in copper-treated soil were more susceptible to oxytetracycline/captan contamination. After the successive soil contamination trials the decline of soil respiration was greater in zinc-polluted soil than in unpolluted soil.

Metallic elements with a specific mass higher than 5 g cm^−3^ and able to form sulphides are commonly called heavy metals. Trace metals would be the more correct term, since the latter is based only on concentration (~0.1% in soil or 100 mg kg^−1^ in dry matter of biological samples) (Leyval et al. 1997). Common anthropogenic sources of trace metal contamination are metal mining and smelting, industry, agriculture (including the application of municipal sewage sludge) and fossil fuel combustion (Kabata-Pendias and Pendias 2001). Like all elements, metals are not biodegradable and once incorporated into the soil they remain for very long periods, up to thousands of years (Kabata-Pendias and Pendias 2001).

Because of their small size, microorganisms have a high surface-area-to-volume ratio and thus a large contact area which can interact with contaminants in the surrounding environment (Ledin 2000). Soil microbial properties are often used as indicators of soil quality and contamination (Hill et al. 2000; Renella et al. 2004). Reduced microbial activity and biomass and/or changes in the microbial community structure have been reported frequently in studies of metal-contaminated soils (Frostergård et al. 1996; Kelly et al. 1999), but in chronically polluted soils the microorganisms can adapt to even high concentrations of heavy metals through various adaptive mechanisms (Díaz-Raviña et al. 1994; Piotrowska-Seget et al. 2005).

Oxytetracycline and captan are compounds typically used to selectively inhibit soil bacteria or fungi. The basis of this method is to use selective chemicals, a bactericide (oxytetracycline) and fungicide (captan), to assess the activities of soil bacteria and fungi, respectively (Lin and Brooks 1999, Bailey et al. 2002). Inhibition of respiration in different (unpolluted) soils ranged from 29% to 59% when these chemicals were applied simultaneously at an appropriate concentration (Lin and Brooks 1999). The resistance of soil bacteria to trace metals and antibiotics is associated with a mechanism of exchange of plasmids containing both antibiotic- and metal-resistance determinants—genes (Baker-Austin et al. 2006).

The proper functioning of a soil microbial community depends on its capacity to decompose dead organic matter; hence, the effects of stressors are commonly measured as changes in total microbial respiration. Respiration reflects decomposition of organic matter, a major ecological process in soil (Tobor-Kapłon et al. 2006). Respiration can be quantified by measuring the amount of carbon dioxide produced by microorganisms per unit of time. It is a useful measure of soil biota activity. Soil microbial communities are presumed to possess high functional redundancy, so it is expected that if the community structure is altered it will not necessarily be reflected in total microbial activity or biomass, as more resistant species replace the more sensitive ones (Nannipieri et al. 2003). Although the soil respiration rate is not an especially sensitive benchmark, it is widely used to detect the influence of various perturbations of soil functional processes (Gong et al. 2000; Borken et al. 2002; Stefanowicz et al. 2008).

It is generally accepted that higher biological diversity enhances community resilience and resistance, understood as its ability to resist and recover from a perturbation (MacArthur 1955; Steiner et al. 2006). Resilience is a component of stability and is defined as the ability of a community to recover over time after a stress, while resistance means its ability to withstand immediate stress effects (Seybold et al. 1999). Metal-tolerant microbial populations associated with metal-contaminated soils may have lower genetic diversity; this should make the community less able to adapt to successive bouts of contamination. The purpose of this study was to compare the toxic effects of applying copper and a mixture of oxytetracycline and captan on the respiration rate of microorganisms originating from unpolluted soil and from soil chronically polluted with zinc. The zinc-polluted soil organisms were expected to exhibit higher vulnerability to repeated contamination than those of unpolluted soil.

## Materials and Methods

Humus samples were collected from forest sites in southern Poland near Olkusz in the vicinity of former zinc and lead smelters. Mining activity in the area dates to the Middle Ages; large-scale industry in the Olkusz region started in the 1970s. The effects of chronic metal pollution on different levels of biological organisation have been intensively studied there for many decades (Wóycicki 1913; Dobrzańska 1955; Laskowski and Maryański 1993; Gdula-Argasińska et al. 2004; Klimek and Niklińska 2007; Stefanowicz et al. 2008; Grześ 2009).

Ten samples of polluted (P) soil were collected close to Olkusz (N 50°18′; E 19°30′) and ten samples of unpolluted soil (UP) were collected near Nakło (N 50°41′; E 19°43′). Both sites are covered by Scots pine forest (*Pinus sylvestris*) with admixture of birch (*Betula pendula*). The collected soil samples were immediately sieved (1 cm) to remove the green parts of plants, stocks, stones and roots, thoroughly hand-mixed, and transported to the laboratory. The samples were stored for 2 weeks in laboratory conditions prior to microbial analysis (field moisture, 10°C).

At this time the physicochemical parameters of the soils were measured: water content (after 24 h drying at 105°C), organic matter content (loss on ignition after 24 h at 550°C), water holding capacity (WHC, standard gravimetric method) and pH in H_2_O (1:10 w:v). The total concentrations of heavy metals (Zn_t_, Cu_t_) were measured after wet digestion of dried and ground subsamples in suprapure concentrated nitric acid (Sigma-Aldrich). The water-soluble metal concentrations (Zn_w_, Cu_w_) were extracted by shaking samples with deionized water (pH 5.5). Zn and Cu concentrations (total and water-soluble) were measured by flame or graphite furnace atomic absorption spectrometry (Perkin-Elmer Analyst 800). All chemical data are expressed per unit of dry weight (dw) of soil.

Samples of P and UP soils (equivalents of 7 g dw) were placed in plastic beakers. Both soils were contaminated with copper chloride at copper concentrations of 500, 1,500 and 4,500 mg kg^−1^ dw soil; subsamples without copper added were the controls for both soil types. Four replicates for each treatment were made at this stage (32 beakers total). The soil samples were incubated for 4 weeks in constant laboratory conditions at 22 ± 1°C and 75% WHC in climate chambers. Sample moisture was kept constant by adjusting daily with deionized water.

After that period, substrate-induced respiration (SIR) was measured in the soil samples after the addition of glucose solution (Rühling and Tyler 1973). Then in the subsequent soil contamination trial a mixture of oxytetracycline and captan together with glucose was added to half of the soil subsamples. Oxytetracycline (Sigma-Aldrich) can bind to the 30S ribosome sub-unit and inhibit protein synthesis in a range of bacteria, and has little effect on soil fungi (Bailey et al. 2002). Captan (Organika-Azot, Jaworzno, Poland) is a broad-spectrum thiophosgene fungicide which has little effect on soil bacteria (Bailey et al. 2002). Both chemicals were added to the soil at concentrations of 10 mg g^−1^ dw soil together with glucose, gently mixed with the soil, and incubated for 6 h at 22 ± 1°C.

The respiration rate of each sample was measured by trapping CO_2_ in 5 mL 0.2 M NaOH in airtight glass jars. After 6 h incubation of the soil samples in the tightly closed jars, 2 mL BaCl_2_ was added to the NaOH solution, and the excess of sodium hydroxide was titrated with 0.1 M HCl in the presence of phenolphthalein as coloured indicator. The blanks were empty jars randomly distributed among the others. The respiration rate for each sample is expressed as mM CO_2_ per kg organic matter per 24 h.

Multifactor ANOVA was applied to test the significance of the effects of soil type (P, UP), copper contamination (control, 500, 1,500, 4,500 mg kg^−1^ dw) and the organic pollutants (oxytetracycline and captan or their absence), and the interactions between the tested factors, on the soil respiration rate. Statistical analyses employed Statgraphics Centurion (StatPoint Technologies Inc., Warrenton VA, USA).

## Results and Discussion

The results of physicochemical soil analyses are given in Table 1. The total and soluble zinc concentrations in polluted soil (P) were higher than in unpolluted soil (UP) by more than an order of magnitude (Table 1). Total copper was also higher in P than in UP soil, but the soluble Cu concentrations in P and UP soil were similar and rather low. While zinc remains the major soil pollutant in the Olkusz region, the soil there is highly polluted with other heavy metals as well. Total concentrations of other heavy metals in soil close to the studied polluted site were reported recently: Pb ~ 1,200 mg kg^−1^ dw, Cd ~ 39 mg kg^−1^ dw, Ni ~ 14 mg kg^−1^ dw and Tl ~ 19 mg kg^−1^ dw (see forest transect in Stefanowicz et al. 2008).

**Table 1:** Chemical characteristic of zinc-polluted (P) and unpolluted (UP) soil

Soil kind	Statistics	OM	WHC	pH	Zn_t_	Zn_w_	Cu_t_	Cu_w_
UP	X	57.0	398.0	4.68	140.70	3.83	39.86	0.29
	SD	10.1	92.4	0.24	42.23	2.92	6.72	0.04
	V	0.2	0.23	0.05	0.30	0.76	0.17	0.13
P	X	54.0	448.0	5.26	5,018.00	82.30	83.37	0.24
	SD	8.0	82.0	0.35	666.30	12.45	22.14	0.09
	V	0.2	0.18	0.07	0.13	0.15	0.27	0.37

Values are means (X) with standard deviation (SD) and variance coefficient (V) (n = 10). Organic matter content (OM) and water holding capacity (WHC) are given as % of dry weight; total and water-soluble zinc and copper concentrations (Zn_t_, Zn_w_, Cu_t_ and Cu_w_) are given as mg kg^−1^ dw. Standard deviations are given in the same units as the given parameter

Soil pH can influence the solubility of metals, and metal contamination typically reduces soil pH (Speir et al. 1999; Boivin et al. 2006). Interestingly, in my study the pH of polluted soil was higher than that of the unpolluted soil; factors other than metal pollution must have affected their pH.

**Fig. 1:**
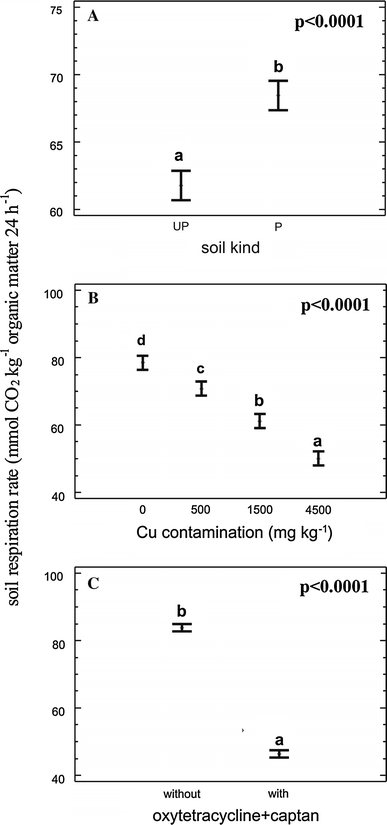
Effects of soil type (**a**), copper contamination (**b**) and combined oxytetracycline/captan contamination (**c**) on soil respiration rate. *Central points* indicate sample means, and *error bars* indicate 95% Tukey HSD intervals. *Different letters above bars* indicate significant differences

The measured soil respiration rates here ranged from 30.07 mmol CO_2_ kg^−1^ OM 24 h^−1^ (P soil contaminated with 4,500 mg Cu^2+^ kg dw soil and afterwards with oxytetracycline and captan) to 115.89 mmol CO_2_ kg^−1^ OM 24 h^−1^ (controls of P soil, Table 2). Multifactor ANOVA for respiration rate showed a significant effect of soil type (F = 40.73, *p* < 0.0001, Fig. 1a), Cu contamination (F = 137.14, *p* < 0.0001, Fig. 1b) and combined oxytetracycline/captan application (F = 1,273.13, *p* < 0.0001, Fig. 1c).

**Table 2:** Effects of laboratory Cu contamination and oxytetracycline and captan (O+C) contamination on the respiration rate of unpolluted (UP) and chronically zinc-polluted (P) soils

Soil kind	Cu treatment mg kg^−1^ dw	O+C treatment	Respiration rate mmol CO_2_ kg^−1^ OM 24 h^−1^			% of control
			X	SD	V	
UP	0	No	**89.23**	0.71	0.01	**100**
	500	No	84.23	1.41	0.02	94
	1,500	No	78.29	2.18	0.03	88
	4,500	No	64.33	2.82	0.04	72
	0	Yes	51.94	0.69	0.01	58
	500	Yes	46.96	0.11	0.00	53
	1,500	Yes	41.99	0.69	0.02	47
	4,500	Yes	37.13	0.95	0.03	42
P	0	No	**109.45**	9.11	0.08	**100**
	500	No	96.67	3.34	0.03	88
	1,500	No	80.77	0.57	0.01	74
	4,500	No	67.80	0.16	0.00	62
	0	Yes	63.43	4.11	0.06	58
	500	Yes	55.20	1.83	0.03	50
	1,500	Yes	43.34	3.24	0.07	40
	4,500	Yes	31.05	1.38	0.04	28

Values are means (X) with standard deviation (SD) and variance coefficient (V) (n = 2)

Respiration rates of control samples of P and UP soil (not contaminated in laboratory with copper nor oxytetracycline/captan) are given in bold

The zinc-polluted soil had a higher mean respiration rate than the unpolluted soil (Fig. 1a). Soil microorganism activity is affected by various factors. In this study the soil respiration rate is expressed per unit of organic matter, which was determined in each soil sample after the respiration measurements. The result may reflect differences in the proportions of glucose-metabolizing microbes in these soils. The methodology of substrate-induced respiration (SIR) measurement assumes that the proportion of microorganisms able to break down glucose is the same in each soil (Anderson and Domsch 1978), but that may not be true of every soil type. Anderson and Joergensen (1997) showed that the extremely low pH and high organic matter content of many forest soils may confound SIR estimates of microbial biomass. The use of glucose to measure soil respiration accurately may be problematic, but to measure the soil microbial response shortly after contamination it is necessary to use easily degradable carbon compounds. Otherwise, the decay of dead microbial cells may measurably increase the soil respiration rate, as Leita et al. (1995) showed in an experiment with metal-contaminated soil. Other effects possibly concealed in the final results are connected with differences in physicochemical soil properties not measured here, such as content of phosphorus or other limiting chemical elements.

**Fig. 2:**
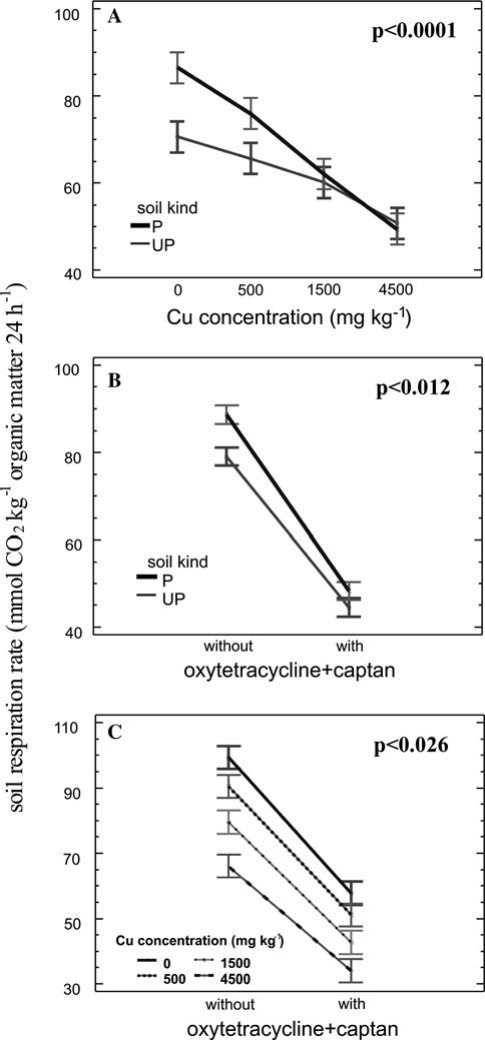
Effects of interactions between soil type and copper contamination (**a**), between soil type and combined oxytetracycline/captan contamination (**b**), and between copper contamination and combined oxytetracycline/captan contamination (**c**), on soil respiration rate. *Central points* indicate sample means, and *error bars* indicate 95% Tukey HSD intervals

Multifactor ANOVA showed that the higher respiration rate in zinc-polluted soil was significant but that the effect was dependent on other factors. There were significant interactions between soil kind and copper contamination (F = 13.91, *p* < 0.0001; Fig. 2a) and between soil type and oxytetracycline/captan treatment (F = 7.90, *p* < 0.012; Fig. 2b). There was also a significant interaction between Cu contamination and the effect of the two organic toxicants (F = 3.90, *p* < 0.026; Fig. 2c). Third-order interactions were not significant and were removed from the model; the lack of such interesting relationships probably is attributable to an insufficient number of replicates, making this experiment a pilot study which will have to be followed up.

Above all, the highly significant interaction between soil kind and copper contamination indicated that in zinc-polluted soil the application of copper caused a larger reduction of the respiration rate than in unpolluted soil (Fig. 2a). Typically, microorganisms in chronically polluted soils exhibit a phenomenon called pollution-induced community tolerance (PICT) (for a review see Blanck 2002). Soil microbial communities can adapt to higher trace metal concentrations in the ambient environment due to selection of more resistant organisms, physiological acclimation and genetic adaptation. This can cause shifts in community composition and can change the functioning of the whole community (Blanck 2002; Almås et al. 2004). Detection of PICT gives a second measure of the microbial community’s response to contamination (Díaz-Raviña et al. 1994).

Separating the direct effects of a metal from the effects of other soil physicochemical properties on soil processes in metal-contaminated soil states a strong argument for the operation of PICT in a community. Kelly et al. (1999), for example, showed the development of Zn resistance in microbial communities in Zn-contaminated soil (4,660 mg kg^−1^ dw) but not in control treatments or in pH-control treatments.

In my study the interaction between soil type and laboratory copper contamination indicated the opposite effect, possibly due to differences in methodology. I measured the effect of copper on the soil respiration rate as late as 4 weeks after the copper treatment was applied; while PICT must be detected after a very short interval, before changes in community structure occur which could skew the result (Blanck 2002). The purpose of my study was not to detect PICT but rather to show that several successive contamination events can cause a gradual decrease of the soil microbial respiration rate, and that this decrease will be stronger in chronically polluted soil.

Copper contamination as well as the oxytetracycline/captan treatment reduced the majority of soil respiration rates, but to different degrees in the two soil types (Table 2). The mean soil respiration rate in treatments with the highest Cu concentration and oxytetracycline/captan was 42% of the control rate for UP soil, but only 28% for P soil. The P soil controls had a higher respiration rate than UP soil controls, but after Cu treatment the decrease of the respiration rate was larger for P soil than for UP soil (Fig. 2a). Similarly, after the combined oxytetracycline/captan treatment the decrease was greater for P soil than for UP soil (Fig. 2b). The relationships between toxicants were quite interesting (Fig. 2c): the difference most visible on the plot is between Cu-free samples and samples contaminated with Cu at 500 mg kg^−1^ dw and higher, for the joint effect of oxytetracycline and captan on the respiration rate. The decrease was greater for Cu-free soil than for Cu-contaminated soil. This observation might be interpreted in terms of PICT. According to Blanck (2002), PICT may be observed in short-term tests with recurrent exposure. In this study the effect was measured 6 h after oxytetracycline/captan were applied.

In these experiments the microbial communities of zinc-polluted soil proved more susceptible to repeated stress. Presumably the microbial community of the sampled chronically polluted soil had lower genetic diversity and thus experienced a stronger negative effect of the subsequent contamination treatments than the unpolluted soil did, but additional microbial analyses would be needed to confirm this. In such studies the choice of the point in time for quantifying the microbial community’s response is critical. There is an inherent dilemma: it can be argued that short-term tests may be generically less sensitive than tests of longer duration (Blanck 2002). Also, laboratory application of chemicals such as readily soluble metal salts imposes a sudden stress on the microorganisms, and usually is not comparable to field situations.
